# Prenatal treatment with rapamycin restores enhanced hippocampal mGluR-LTD and mushroom spine size in a Down’s syndrome mouse model

**DOI:** 10.1186/s13041-021-00795-6

**Published:** 2021-05-25

**Authors:** Jesús David Urbano-Gámez, Juan José Casañas, Itziar Benito, María Luz Montesinos

**Affiliations:** 1 Departamento de Fisiología Médica Y Biofísica, Universidad de Sevilla, Av. Sánchez-Pizjuán 4, 41009 Sevilla, Spain; 2 Instituto de Biomedicina de Sevilla, IBIS/Hospital Universitario Virgen del Rocío/CSIC/Universidad de Sevilla, Sevilla, Spain; 3 Servicio de Animalario, Hospital Universitario Virgen Macarena (HUVM), 41009 Sevilla, Spain

**Keywords:** mGluR-LTD, mTOR, Dendritic spines, Proteomics, Down syndrome, Synaptoneurosomes, Trisomy 21

## Abstract

Down syndrome (DS) is the most frequent genetic cause of intellectual disability including hippocampal-dependent memory deficits. We have previously reported hippocampal mTOR (mammalian target of rapamycin) hyperactivation, and related plasticity as well as memory deficits in Ts1Cje mice, a DS experimental model. Here we characterize the proteome of hippocampal synaptoneurosomes (SNs) from these mice, and found a predicted alteration of synaptic plasticity pathways, including long term depression (LTD). Accordingly, mGluR-LTD (metabotropic Glutamate Receptor-LTD) is enhanced in the hippocampus of Ts1Cje mice and this is correlated with an increased proportion of a particular category of mushroom spines in hippocampal pyramidal neurons. Remarkably, prenatal treatment of these mice with rapamycin has a positive pharmacological effect on both phenotypes, supporting the therapeutic potential of rapamycin/rapalogs for DS intellectual disability.

**Supplementary Information:**

The online version contains supplementary material available at 10.1186/s13041-021-00795-6.

## Introduction

DS, also known as trisomy 21, is one of the most common causes of intellectual disability. Among other difficulties, DS patients experience learning and memory deficits that evidence hippocampal dysfunctions [1]. We have previously shown that synaptic local translation, a key process for plasticity and hippocampal-dependent memory, is deregulated in the DS mouse model Ts1Cje [2] due to mTOR hyperactivation [3]. Accordingly, mTOR hyperactivation has been also found in subjects with DS [4, 5]. In Ts1Cje mice, mTOR hyperactivation seems to be caused by increased levels of Brain Derived Neurotrophic Factor (BDNF) that saturate the BDNF–TrkB–Akt–mTOR signaling axis [3, 6]. Furthermore, we also found that the BDNF-dependent Long-Term Potentiation (LTP) is abolished in the Ts1Cje hippocampus, and that the specific mTOR inhibitor rapamycin fully restored this type of plasticity [7]. Moreover, we observed that the impaired persistence of long-term memory (LTM) in the Barnes maze of Ts1Cje animals was also restored by rapamycin treatment [7].

The mTOR pathway is known to participate in other forms of plasticity, such as mGluR-LTD. This is mediated by group I metabotropic glutamate receptors and relies on protein translation [8]. mTOR forms two different complexes: mTORC1, which contains, among other components, the defining protein RAPTOR (Regulatory Associated Protein of mTOR), and mTORC2, which contains RICTOR (Rapamycin-Insensitive Companion of TOR). In neurons, mTORC1 is mainly involved in translational control, mitochondrial function and autophagy regulation, whereas mTORC2 regulates actin cytoskeleton (for a review see [9]). It is well known that rapamycin blocks hippocampal mGluR-LTD [10], initially suggesting that protein synthesis mediated by mTORC1 is necessary for this plasticity. Nevertheless, chronic treatment or high concentrations of rapamycin also inhibit mTORC2 [11], and, moreover, it has been recently reported that mTORC2, but not mTORC1, is required for mGluR-LTD [12]. In line with these results, it is well established that inhibiting actin polymerization/depolymerization blocks mGluR-LTD [13] and that dendritic spines elongate in response to mGluR activation, correlating with AMPA receptor (AMPAR) endocytosis [13, 14]. In fact, mGluR activation induces rapid local translation of proteins involved in AMPAR internalization such as Arc/Arg3.1 (Activity Regulated Cytoskeleton-associated protein), OPHN1 (oligophrenin-1), MAP1B (Microtubule Associated Protein 1B) and STEP (Striatal-Enriched Protein Phosphatase) [15]. These proteins are targets of FMRP (Fragile X Mental Retardation Protein, encoded by the *FMR1* gene), a key regulator of local translation. Since mGluR-LTD is enhanced in *FMR1* knockout mice, it has been proposed that FMRP serves as a brake on mGluR-stimulated protein synthesis (for a review see [16]). Moreover, it has been shown that hippocampal mGluR-LTD requires the rapid synthesis and degradation of FMRP [17]. Despite the extensive work, the definitive comprehension of the signaling pathways that contribute to protein synthesis necessary for mGluR-LTD remains elusive although roles for mTORC1 and ERK (Extracellular signal-regulated kinase) have been proposed [16].

To gain insight into the signaling pathways relevant for synaptic plasticity altered in Ts1Cje, we have characterized the proteome of hippocampal SNs from wild-type (WT) and Ts1Cje mice by iTRAQ (isobaric tag for relative and absolute quantitation). Interestingly, mitochondrial function, calcium signaling and, remarkably, synaptic plasticity pathways, including LTD, were predicted to be altered in trisomic mice. Accordingly, we have found that the hippocampal mGluR-LTD is enhanced in Ts1Cje animals. Additionally, we have evaluated the effect of rapamycin on dendritic spine density and morphology. We found that prenatal treatment with rapamycin did not recover the decreased density of dendritic spines in Ts1Cje offspring but, interestingly, recovered the alterations observed in mushroom spine size. Strikingly, prenatal rapamycin treatment also had a recovery effect on the mGluR-LTD in Ts1Cje hippocampus. Together, these results extend the evidence that supports the possible benefits of rapamycin for synaptic plasticity in the context of DS.

## Materials and methods

### Animals

Partial trisomic Ts1Cje mice [2] were purchased from Jackson Laboratories (RRID:IMSR_JAX:004861) and, as recommended by the supplier, were maintained on a mixed genetic background for improving strain viability, by backcrossing Ts1Cje males to B6C3F1 hybrid females (supplied by Charles River, RRID:IMSR_CRL:31). The Ts1Cje mice harbour two copies of the WT murine chromosome 16 (MMU16) and an additional copy of distal MMU16 (containing 96 genes) translocated onto MMU12 [2]. Sets of WT males and Ts1Cje littermates were used in the experiments. A total number of 48 animals (22 WT and 26 Ts1Cje) were used. No randomization was performed to allocate subjects in the study. Thus, they were arbitrarily allocated to the specific treatments or procedures. Blind animals homozygous for the retinal degeneration mutation *Pde6b*^rd1^, which segregates in the Ts1Cje colony [2], were not used. Experiments were carried out according to the European Union directive for the use of laboratory animals. All the protocols were approved by the Regional Government (Junta de Andalucía, Spain) Ethical Committee (Ref. No. 24/05/2016/097). This study was not pre-registered. Mice were housed in cages and subjected to a normal 12 h light–12 h dark cycle. In the case of adult and juvenile (P21–P30; P, postnatal day) mice, 4 animals per cage, with food and water ad libitum, were housed. P18 mice, used in Golgi experiments, were housed with their mother.

Animals were genotyped following the protocols recommended by Jackson Laboratories. For determining partial trisomy, the neomycin resistance generic primers oIMR0013 (5ʹ-CTTGGGTGGAGAGGCTATTC) and oIMR0014 (5ʹ-AGGTGAGATGACAGGAGATC), which generate a 280 bp NEO band (only present in trisomic mice), as well as the internal control Tcrd primers oIMR0015 (5ʹ-CAAATGTTGCTTGTCTGGTG) and oIMR0015 (5ʹ-GTCAGTCGAGTGCACAGTTT), which amplify a 200 bp fragment (both in trisomic and WT mice), were used in standard PCR reactions. For detecting the *Pde6b*^*rd1*^ mutation, the oIMR2093 (mutant; 5ʹ-AAGCTAGCTGCAGTAACGCCATTT), oIMR2094 (WT; 5ʹ-ACCTGCATGTGAACCCAGTATTCTATC) and oIMR2095 (common; 5ʹ-CTACAGCCCCTCTCCAAGGTTTATAG) primers were used in standard PCR reactions. These primers produce a 560 bp band in mutants, a 240 bp band in WT, and both in heterozygote animals.

Adult animals were sacrificed by cervical dislocation. P18–P30 animals, used for electrophysiology and Golgi staining experiments, were anaesthetized by intraperitoneal injection of 2% tribromoethanol (Sigma, Ref. T48402) at a dose of 300 mg/kg, and then decapitated. Finally, P0 mice used for primary culture of neurons were directly decapitated.

### Rapamycin treatment

Rapamycin (Selleckchem, Ref. S1039) was dissolved at 0.1 mg/ml in saline buffer containing 1 ethanol (Merck, Ref. 100983), 0.25% Tween^®^80 (Sigma-Aldrich, Ref. P4780) and 0.25% polyethylene glycol 400 (Aldrich, Ref. 202398) [18]. A single intraperitoneal injection of rapamycin (1 mg/kg) was applied to pregnant dams between E15 and E17 as previously described [18]. Control pregnant dams were not injected with vehicle.

### Synaptoneurosomes preparation

SNs were prepared as previously described [19]. Briefly, hippocampi from adult WT or Ts1Cje mice (2–4 month old, 3 mice per group) were rapidly dissected and homogenized in 12 ml of homogenization solution consisting in: 10 mM Hepes (Sigma, Ref. H4034), pH 7.4; 320 mM sucrose (Sigma, Ref. S0389); 1.0 mM MgSO_4_ (Sigma, Ref. M7506); protease inhibitors leupeptin (10 μM), aprotinin (2 μg/ml), and pepstatin (1 μM) (Sigma, Ref. L2884, A1153 and P5318, respectively), with a glass-teflon Dounce homogenizer. The homogenate was centrifuged (1000*g* for 10 min at 4 °C), and the pellet was subjected to an Optiprep discontinuous gradient (9–40% Optiprep; Sigma, Ref. D1556). After centrifugation (16,500*g* for 30 min at 4 °C), the material from the first band (fraction O1) was recovered and subjected to discontinuous Percoll (Sigma, Ref. P1644) gradient (10–25%) centrifugation (32,400*g* for 20 min at 4 °C). The material from the fourth band (fraction 1P4), which is highly enriched in SNs, was recovered, washed with buffer, rapidly frozen and stored at − 80 °C until iTRAQ proteomics.

### iTRAQ labeling and analysis

Protein extraction, iTRAQ labeling and tandem mass spectrometry analysis was carried out at the Instituto de Biomedicina de Sevilla (IBiS) Proteomic Service. Briefly, synaptoneurosomal proteins were extracted using a lysis buffer that contained SDS, supplemented with protease inhibitors (Sigma, Ref. P8340), phosphatase inhibitor cocktails I and II (Sigma, Ref. P2850 and P5726), and benzonase (Sigma, Ref. E8263). After incubation for 1 h, the samples were centrifuged for 15 min at 14,000 rpm in a refrigerated bench-top microfuge. Proteins present in the supernatant were quantified following iTRAQ labeling (AB ScieX, Ref. 4390811) essentially following the manufacturer’s instructions, omitting the protein precipitation step in order to conserve minority proteins. 50 μg of proteins were labeled for each experimental condition in duplicates. Data were analyzed using the Proteome Discoverer 1.4 software (Thermo), setting the False Discovery Rate (FDR) of both proteins and peptides identification to be less than 0.01.

### Proteomic analyses

To verify that SNs preparations used in iTRAQ experiments were enriched in synaptic proteins, a PANTHER (Protein ANalysis THrough Evolutionary Relationships; version 16.0) analysis was carried out [20]. An overrepresentation test was performed, selecting the entire Mus musculus genome (21,988 proteins) and the PANTHER GO-Slim Cellular Component annotation data set. Bonferroni correction for multiple testing was applied.

Ingenuity Pathway Analysis (IPA, Fall release September 2019) was performed for both up- and downregulated proteins, considering a cutoff of 1.2-fold. This cutoff was established by analyzing the protein level variations observed in the WT sample replicate and determining the boundaries within the 80% best fitting replicate data.

The Ingenuity Knowledge Base (genes only) set was used as reference. All the molecules and/or relationships included in the analysis were experimentally observed, either in the mouse, rat, human nervous system tissue or neural (astrocytes or neurons) cells. The IPA software generates a list of significantly affected canonical pathways based on two parameters, the p-value and the Z-score. The p-value, calculated with the right-tailed Fisher Exact Test, is a measure of the probability that a particular pathway were enriched in the set of deregulated proteins due to random chance. To enhance the stringency of the analysis, we considered only pathways with a p-value ≤ 0.005 (i.e., − log_10_ (p-value) ≥ 2.3). Considering the protein level changes observed in the set of deregulated proteins, the canonical pathways identified are predicted to be either activated or inhibited applying the IPA Z-score algorithm. A positive Z-score value indicates that a pathway is predicted to be activated, and a negative Z-score indicates its inhibition. Canonical pathways which are not eligible for activity analysis by the IPA are marked as N/A.

### Antibodies

We used anti-FMRP (Abcam; Ref. ab69815, RRID:AB_1209562), anti-MAP2 (Merck Millipore; Ref. MAB378, RRID:AB_94967), anti-LC3B (Cell Signaling; Ref. #2775, RRID:AB_915950), and anti-TOMM20 (Novus; Ref. NBP1-81556, RRID:AB_11003249) as primary antibodies, while horseradish peroxidase (HRP)-conjugated anti-rabbit (Promega; Cat·W4011, RRID:AB_430833), Alexa Fluor 555 goat anti-rabbit (Thermo Fisher Scientific; Cat# A-21429, RRID:AB_2535850), and Alexa Fluor 488 goat anti-mouse (Thermo Fisher Scientific; Cat# A-11001, RRID:AB_2534069) were used as secondary antibodies.

### Western blot

Western blots were performed as previously described [21]. Adult (4–5 month-old) male mice were used. Briefly, total hippocampal protein extracts were prepared for each individual by mechanical tissue homogenization in extraction buffer containing: 50 mM Tris (Sigma, Ref. 93352) buffer (pH 7.4), 0.5% Triton X-100 (Sigma, Ref. T8787), 150 mM NaCl (Sigma-Aldrich, Ref. S7653), 1 mM EDTA (Sigma, Ref. E5134), 3% SDS (Sigma, Ref. 05030), protease (Sigma, Ref. P8340) and phosphatase inhibitor cocktails (Sigma, Ref. P5726 and P0044). Protein extracts were resolved by SDS-PAGE on Mini-PROTEAN^®^ TGX Stain-Free™ precast gels (BioRad, Ref. 1704156). Proteins were transferred to polyvinylidene difluoride (PVDF) membranes (Trans-blot^®^ Turbo™ Transfer Pack, Ref. 1704156). Membranes were incubated with blocking solution (1% non fat dry milk in TBS-T buffer; TBS-T buffer is 20 mM Tris buffer [pH 7.5], 150 mM NaCl, and 0.1% Tween^®^20) for 1 h at RT, then incubated overnight at 4 °C with the appropriate primary antibody (dilution 1:1,000). After washing with TBS-T, the membranes were incubated with the HPR-conjugated secondary antibodies diluted in blocking solution (dilution 1:20,000 for FMRP immunoblot, 1:5,000 for LC3B immunoblot, and 1:2,000 for TOMM20) for 1 h at RT. HRP-conjugated secondary antibodies were detected with the WesternBright Quantum HRP Substrate (Advansta, Ref. K-12042-D10) and chemiluminiscence measured on a ChemiDoc XRS (BioRad) imager.

### Immunocytochemistry

Hippocampal cultures from P0 WT or Ts1Cje littermates were established as previously described [22]. Briefly, hippocampi from 5 WT and 5 Ts1Cje mice were dissected in HBSS medium (Gibco, Ref. 14175-095) and mechanically dissociated in DB1 culture medium (Biowest, Ref. L0104) after treatment with 0.2% trypsin (Gibco, Ref. 15090-046) for 10 min. Cells were cultured in Neurobasal A (Gibco, Ref. 100888-022) supplemented with B27 (Gibco, Ref. 17504-044), Glutamax (Gibco, Ref. 35050-061) and penicillin/streptomycin (Gibco, Ref. 15140-122). To inhibit glial growth, 0.3 mM 5-fluoro-2ʹ-deoxyuridine (Sigma-Aldrich, Ref. 856657) and 0.8 mM uridine (Sigma-Aldrich, Ref. U3003) were added after 48 h in culture. After 14 days in vitro (DIV), cells were fixed with 4% paraformaldehyde (PFA, Sigma-Aldrich, Ref. P6148) in phosphate-buffered saline (PBS, Panreac Applichem, Ref. A9177) and subjected to dual immunocytochemistry using antibodies against FMRP (1:250 dilution) and MAP2 (1:1,000 dilution). Thus, after fixation, cells were washed 3 times with PBS and incubated with blocking solution (0.1% Triton X-100, and 10% fetal bovine serum in PBS) for 1 h at RT. Then, primary antibodies, diluted in blocking solution, were applied overnight at 4 °C. After washing 3 times with PBS for 10 min, cells were incubated with the corresponding secondary antibodies (dilution 1:1,000) for 1 h at RT, then washed in PBS. Confocal microscopy images were acquired and processed as previously described [21]. The mean pixel intensity for the corresponding immunofluorescent FMRP signal in dendrites was determined (in arbitrary units, a.u.) using a Matlab (Mathworks) routine previously established [3].

### Electrophysiological recordings

Hippocampal slices were prepared from P21–P30 WT and Ts1Cje mice. Since we intended to correlate electrophysiological and behavioural results, and previous behavioural experiments performed in the laboratory gave a high variability when using females, only male mice were used, for the sake of reproducibility. Mice were anesthetized and brains were removed in an ice-cold artificial cerebrospinal fluid with partial substitution of Na with sucrose (ACSFs) with constant flux of carbogen (5% CO_2_; rest O_2_; H_2_O < 5 ppm). ACSFs composition was: 2.5 mM KCl (Sigma, Ref. P9541); 1.25 mM NaH_2_PO_4_ (2H_2_O) (Sigma, Ref. P71505); 26 mM NaHCO_3_ (Sigma, Ref. S5761); 25 mM glucose (Sigma, Ref. G5400); 0.5 mM CaCl_2_ (2H_2_O) (Sigma, Ref. 223506); 4 mM MgSO_4_ (7H_2_O) (Sigma, Ref. 63138); 185 mM sucrose (Sigma, Ref. P84097). Brains were positioned in the cutting chamber over a thin film of ethyl cyanoacrylate and were submerged in cold ACSFs. 350 µm horizontal slices were obtained using a vibratome (Vibratome 3000, Sectioning System) and both hippocampi were isolated in each slice preserving entorhinal cortex adjacent to the hippocampal formation. Slices were incubated in ACSF (126 mM NaCl; 3 mM KCl; 1.3 mM MgSO_4_ (7H_2_O); 2 mM CaCl_2_ (2H_2_O); 1.25 mM NaH_2_PO_4_ (2H_2_O); 24 mM NaHCO_3_; 10 mM glucose) for 30 min at 34 °C. Then, slices were incubated for at least 2 h in ACSF at room temperature (RT; 22–25 °C) before recording, keeping constant the flux of carbogen. Slices were transferred to a 3D-MEA chamber (MultiChannel Systems, Ref. 60-3DMEA200/12/50iR-gr) and stayed at least 10 min before stimulation with a 2 ml/min flux rate of ACSF. All the registers were performed at RT. The experimenter was blind to the genotype and treatment of the animals during experimentation and data analysis.

The 3D-MEA devices have 59 conical TiN electrodes with 12 µm diameter (100 µm in the base of the electrode) and 50 µm high, distributed in an 8 × 8 matrix with an internal reference electrode at the position 15 (column 1, row 5). The inter-electrode distance is 200 µm. The isolation material used for these devices’ circuitry is SiN and the base is made out of glass.

Slices were stimulated at the Schaffer collateral pathway in the CA1 region of hippocampus using a biphasic square pulse (negative phase-positive phase; 100 ms per phase) at 0.0167 Hz (1 stimulus per minute). Slices showing field excitatory postsynaptic potential (fEPSP) amplitude lower than 100 µV for test stimulation (−/+ 1750 mV) in the CA1 region were discarded. Input/output (I/O) curve was performed reaching the limits of the system (750–4000 mV; 3 stimuli per amplitude) and baseline was established at 60% of the highest response obtained at the maximum voltage applied. After baseline stabilization, 100 µM (S)-3,5-Dihydroxyphenylglycine (DHPG; Sigma-Aldrich, Ref. D-3689) in ACSF was bath applied at a flow rate of 2 ml/min for 5 min. DHPG-long term depression (DHPG-LTD) was recorded for 1 h after treatment. In order to determine the synaptic efficacy, the slope of the initial fEPSP curve was measured in the segment comprised between the 10–90% of the curve amplitude. Recordings were acquired at 10,000 Hz.

Electrophysiological results are presented as the mean ± SEM (standard error of the mean) normalized to the baseline slope mean. For statistical analysis, a Student’s t-test was performed for compared experimental groups. Normalized slope at the minute 60 after DHPG treatment initiation was used for comparisons.

### Golgi staining and spine morphology analysis

WT and Ts1Cje P18 mice were anesthetized and both hemispheres were separated, and the cerebellum was removed. A FD Rapid Golgi stain kit (FD NeuroTechnologies INC, Ref. PK401A) was used following the manufacturer guidelines. After the staining process, 100 µm coronal slices were prepared using a cryostat (Leica CM1950). Slices were deposited over Menzel-Gläser Superfrost PLUS microscope slides (ThermoScientific, Ref. J1800AMNZ) and covered with a thin gelatine coat (Gelatine Gold; Panreac DC, Ref. 251336).

Secondary dendrites images were acquired from the hippocampal CA1 stratum radiatum region, at a 1,600 × 1,200 pixel resolution, using an Olympus BX61 microscope (CellSens software). For spine morphology analysis, image acquisition was made using an UPlanSApo60x/1.35 oil objective, with a 10× ocular and an additional 2× magnification. For spine density analysis, images were acquired using an UPlanApo40x/0.85 air objective, with a 10× ocular, and an additional 2× magnification. To determine dendritic spine density, spines were manually counted, and the branch longitude was measured using ImageJ software. For spine morphology analysis, spines perimeters were manually defined, and the head diameter was measured using ImageJ software. The experimenter was blind to sample genotype and treatment during experiment and data analysis.

### Statistical analysis

Predetermined sample calculation was not performed. Sample size was determined based on published studies in the field or in animal availability. The quantitative data are presented as the means and SEM. No test for outliers was performed. All data points were included. The GraphPad Prism software (version 5.00) was used. D’Agostino and Pearson omnibus normality test was first performed, in order to apply either a Student’s t-test or a Mann–Whitney rank sum test for mean comparisons. p values are indicated through the text and figure legends.

## Results

### Proteomic analysis of hippocampal SNs from Ts1Cje mice

In a previous work we found that Ts1Cje mice showed impaired BDNF-LTP, which was restored by the mTOR inhibitor rapamycin [7]. In order to identify synaptic differences that could account for plasticity and memory deficits in Ts1Cje mice we performed a proteomic analysis of hippocampal SNs isolated from WT and Ts1Cje mice.

Protein samples were subjected iTRAQ proteomics. Thus, from each SNs sample, duplicates were labeled, and the relative amount of each detected protein was referenced to one of the WT samples (Additional file 1: Table S1). 1,890 proteins were identified by at least two unique peptides (Additional file 1: Table S2). Proteins not detected in all the samples (4 proteins) as well as technical replicates with CV% > 30% (19 proteins) were removed. The geometric mean of the relative protein amount was calculated (Additional file 1: Table S3) and used in the subsequent analysis by IPA software.

**Fig. 1:**
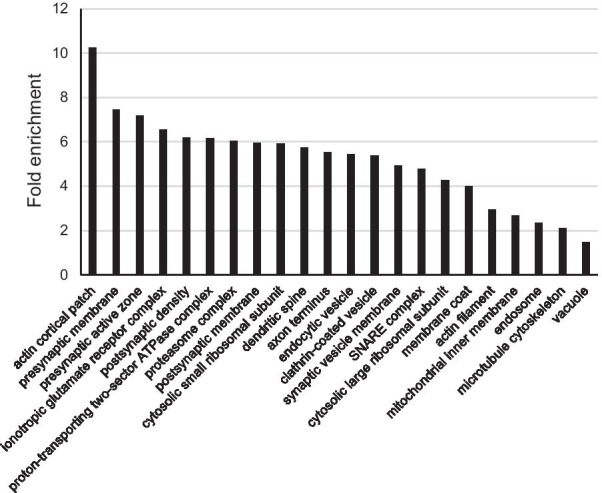
PANTHER overrepresentation test of proteins identified by iTRAQ in WT and Ts1Cje SNs. The cellular components overrepresented in SNs, compared to the complete mouse genome, are shown. For sake of clarity, only the most specific subclasses of the PANTHER Go-Slim Cellular Component annotation data set are included in the figure. Complete analysis is reported in Additional file 1: Table S4. The fold enrichment for each cellular component is indicated

To verify that the detected proteins were mainly synaptic, the list of proteins in Additional file 1: Table S3 was subjected to a PANTHER overrepresentation test [20]. The PANTHER Go-Slim Cellular Component annotation dataset was selected. Thus, sets of related proteins overrepresented into the SNs, compared with the complete mouse genome, were identified. A high enrichment in pre- and post-synaptic proteins was evidenced (Fig. 1 and Additional file 1: Table S4), confirming the quality of the SNs preparations.

From the 1,867 proteins initially included in the IPA, 12 resulted to be unmapped, and from the 1,855 mapped identities, IPA considered 1,824 of them as “analysis-ready”.

Considering a 1.2-fold cutoff (see “Materials and methods”), 116 proteins were found to be deregulated (108 up- and 8 down-regulated) in Ts1Cje SNs compared to WT SNs (Additional file 2: Table S5).

**Fig. 2:**
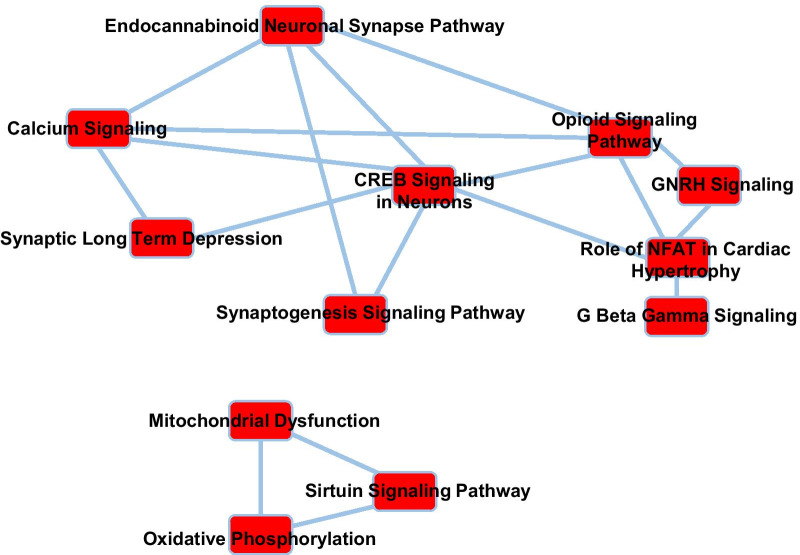
Canonical IPA pathways affected in Ts1Cje SNs. The most significant canonical pathways identified by IPA among the altered proteins in Ts1Cje SNs are shown. Overlapping pathways sharing at least 7 proteins are connected by solid lines

**Fig. 3:**
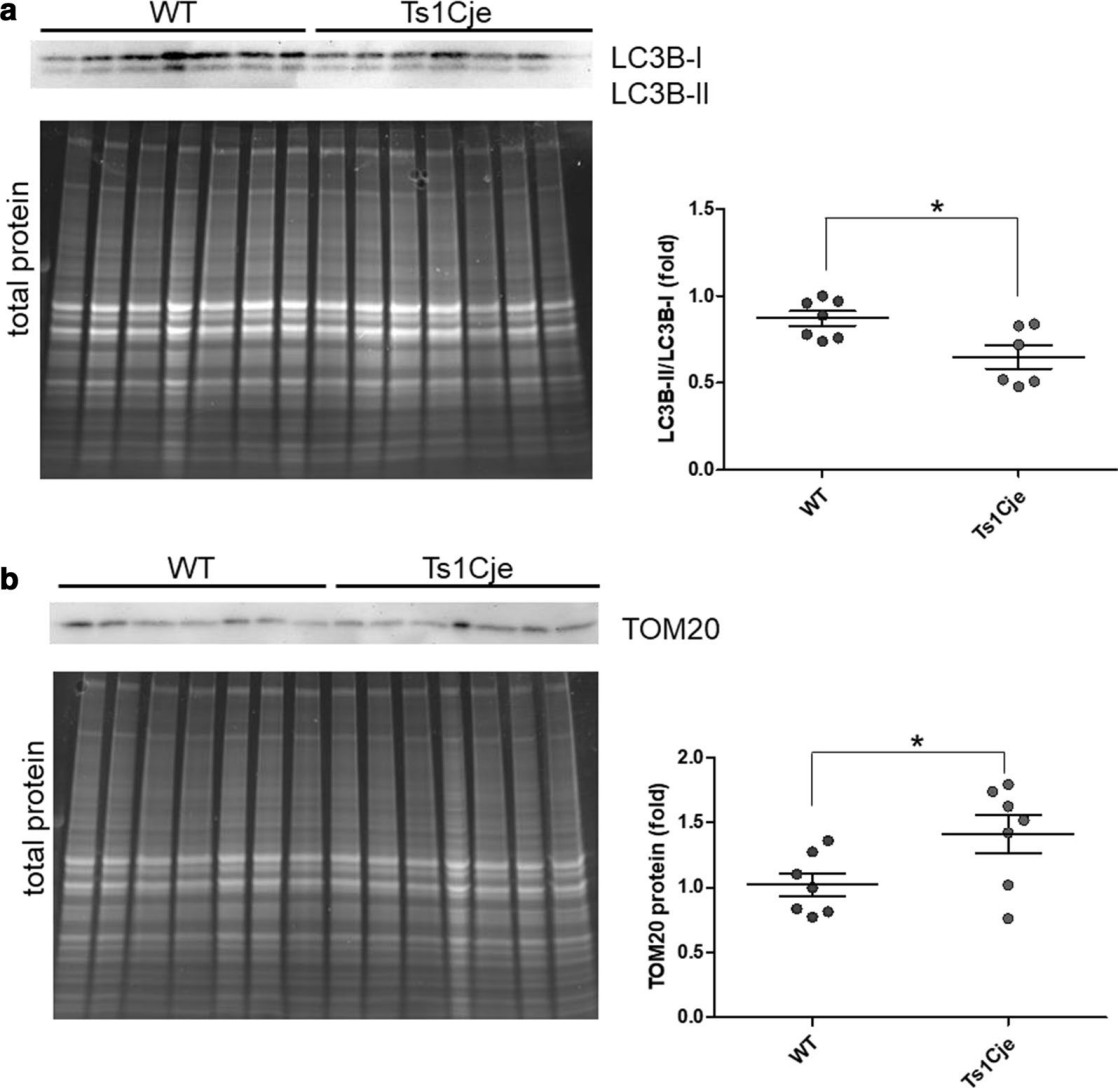
Quantification of LC3B-II and TOM20 proteins in WT and Ts1Cje hippocampus. Hippocampal proteins from WT and Ts1Cje mice pairs were analyzed in Western blot with anti-LC3B or anti-TOM20 antibody. **A** LC3B western blot showing WT and Ts1Cje littermate pairs analyzed and total protein loaded. The ratio LC3B-II/LC3B-I is shown as the mean ± SEM (WT: 0.8714 ± 0.04154; Ts1Cje: 0.6500 ± 0.06802; p = 0.0152, Student’s t-test, n = 7 for WT and n = 6 for Ts1Cje). **B** TOM20 western blot showing WT and Ts1Cje littermate pairs analyzed and total protein loaded. The signals were normalized to the corresponding total protein loaded and the mean ± SEM values are shown (WT: 1.024 ± 0.08799; Ts1Cje: 1.412 ± 0.1452; p = 0.0414, Student’s t-test, n = 7 animals per genotype)

Attending to the Fischer p-value, IPA revealed that, the mitochondrial dysfunction canonical pathway, and the partially overlapping oxidative phosphorylation and sirtuin signaling pathways were among the most significantly enriched in the set of proteins affected in Ts1Cje SNs (Fig. 2, Table 1 and Additional file 2: Table S6). Accordingly, mitochondrial alterations have been described in DS and DS mouse models [23–25]. In fact, it has been recently shown that reduced autophagy/mitophagy due to mTOR hyperactivation produces damaged mitochondria accumulation in DS fibroblasts [26]. In agreement with these data, the levels of the B-II isoform of Microtubule-associated protein 1A/1B-light chain 3 (LC3B-II), a marker of autophagy, are also reduced in Ts1Cje hippocampus (Fig. 3A). Moreover, the levels of TOM20 (Translocase of Outer Mitochondrial membrane 20), a marker of mitochondrial mass, were increased in the Ts1Cje hippocampus (Fig. 3B).

**Table 1:** Proteins involved in mitochondrial pathways affected in Ts1Cje SNs

Symbol	Entrez gene name	ID	Ts1Cje	Pathway
APP	Amyloid beta precursor protein	P12023	1.246	Mitochondrial dysfunction Sirtuin signaling
ATP5MF	ATP synthase membrane subunit f	P56135	1.925	Mitochondrial dysfunction Oxidative phosphorylation
ATP5PD	ATP synthase peripheral stalk subunit d	Q9DCX2	1.523	Mitochondrial dysfunction Oxidative phosphorylation
ATP5PF	ATP synthase peripheral stalk subunit F6	P97450	1.907	Mitochondrial dysfunction Oxidative phosphorylation Sirtuin signaling
COX4I1	Cytochrome *c* oxidase subunit 4I1	P19783	1.818	Mitochondrial dysfunction Oxidative phosphorylation
COX5A	Cytochrome *c* oxidase subunit 5A	P12787	1.889	Mitochondrial dysfunction Oxidative phosphorylation
Cox6c	Cytochrome *c* oxidase subunit 6C	Q9CPQ1	2.119	Mitochondrial dysfunction Oxidative phosphorylation
CYC1	Cytochrome *c*1	Q9D0M3	1.728	Mitochondrial dysfunction Oxidative phosphorylation Sirtuin signaling
CYCS	Cytochrome *c*. somatic	P62897	1.976	Mitochondrial dysfunction Oxidative phosphorylation
GPD2	Glycerol-3-phosphate dehydrogenase 2	Q64521	1.549	Mitochondrial dysfunction
MAOB	Monoamine oxidase B	Q8BW75	1.235	Mitochondrial dysfunction
MT-CO2	Cytochrome *c* oxidase subunit II	P00405	2.804	Mitochondrial dysfunction Oxidative phosphorylation
NDUFA2	NADH:ubiquinone oxidoreductase subunit A2	Q9CQ75	1.337	Mitochondrial dysfunction Oxidative phosphorylation Sirtuin signaling
NDUFA4	NDUFA4 mitochondrial complex associated	Q62425	1.331	Mitochondrial dysfunction Oxidative phosphorylation Sirtuin signaling
NDUFA8	NADH:ubiquinone oxidoreductase subunit A8	Q9DCJ5	1.546	Mitochondrial dysfunction Oxidative phosphorylation Sirtuin signaling
NDUFAF1	NADH:ubiquinone oxidoreductase complex assembly factor 1	A2AQ17	1.359	Mitochondrial dysfunction Sirtuin signaling
NDUFS1	NADH:ubiquinone oxidoreductase core subunit S1	Q91VD9	1.813	Mitochondrial dysfunction Oxidative phosphorylation Sirtuin signaling
NDUFS3	NADH:ubiquinone oxidoreductase core subunit S3	Q9DCT2	1.713	Mitochondrial dysfunction Oxidative phosphorylation Sirtuin signaling
NDUFS6	NADH:ubiquinone oxidoreductase subunit S6	P52503	1.368	Mitochondrial dysfunction Oxidative phosphorylation Sirtuin signaling
NDUFV2	NADH:ubiquinone oxidoreductase core subunit V2	Q9D6J6	1.487	Mitochondrial dysfunction Oxidative phosphorylation Sirtuin signaling
SLC25A4	Solute carrier family 25 member 4	P48962	1.591	Sirtuin signaling
UQCRB	Ubiquinol-cytochrome *c* reductase binding protein	Q9CQB4	1.928	Mitochondrial dysfunction Oxidative phosphorylation
UQCRC1	Ubiquinol-cytochrome *c* reductase core protein 1	Q9CZ13	1.687	Mitochondrial dysfunction Oxidative phosphorylation
UQCRFS1	Ubiquinol-cytochrome *c* reductase. Rieske iron-sulfur polypeptide 1	Q9CR68	3.192	Mitochondrial dysfunction Oxidative phosphorylation Sirtuin signaling
UQCRQ	Ubiquinol-cytochrome *c* reductase complex III subunit VII	Q9CQ69	2.522	Mitochondrial dysfunction Oxidative phosphorylation
VDAC1	Voltage dependent anion channel 1	Q60932	1.762	Mitochondrial dysfunction Sirtuin signaling
VDAC2	Voltage dependent anion channel 2	Q60930	2.043	Mitochondrial dysfunction Sirtuin signaling
WRN	Werner syndrome RecQ like helicase	O09053	− 1.341	Sirtuin signaling

The relative amounts (fold change, compared to WT SNs) of these proteins in Ts1Cje SNs are shown

Most important for the objectives of this work, we found that some key synaptic plasticity related pathways, including calcium signaling, CREB signaling, the endocannabinoid neuronal synapse pathway, the glutamate receptor signaling, and the synaptic LTD, were also significantly affected in Ts1Cje SNs (Fig. 2, Table 2 and Additional file 2: Table S6). In fact, the Z-score analysis of the data predicted increased activity of these pathways in Ts1Cje SNs (Additional file 2: Table S7).

**Table 2:** Proteins involved in synaptic plasticity pathways affected in Ts1Cje SNs

Symbol	Entrez gene name	ID	Ts1Cje	Pathway
ADCY9	Adenylate cyclase 9	P51830	1.344	CREB signaling Endocannabinoid neuronal synapse
ATP2A2	ATPase sarcoplasmic/endoplasmic reticulum Ca2+ transporting 2	O55143	1.251	Calcium signaling
ATP2B1	ATPase plasma membrane Ca2+ transporting 1	G5E829	1.329	Calcium signaling
ATP2B2	ATPase plasma membrane Ca2+ transporting 2	F8WHB1	1.268	Calcium signaling
CACNA1A	Calcium voltage-gated channel subunit alpha1 A	P97445	1.314	Calcium signaling CREB signaling Endocannabinoid neuronal synapse Synaptic LTD
CACNA1G	Calcium voltage-gated channel subunit alpha1 G	Q5SUF8	1.474	Calcium signaling CREB signaling Endocannabinoid neuronal synapse Synaptic LTD
CACNA2D1	Calcium voltage-gated channel auxiliary subunit alpha2delta 1	O08532	1.262	Calcium signaling CREB signaling Endocannabinoid neuronal synapse Synaptic LTD
GNB2	G protein subunit beta 2	E9QKR0	1.207	CREB signaling
GNG2	G protein subunit gamma 2	P63213	− 1.212	CREB signaling Endocannabinoid neuronal synapse Glutamate receptor signaling
GRIA2	Glutamate ionotropic receptor AMPA type subunit 2	P23819	1.481	Calcium signaling CREB signaling Endocannabinoid neuronal synapse Glutamate receptor signaling Synaptic LTD
GRIA4	Glutamate ionotropic receptor AMPA type subunit 4	Q9Z2W8	− 1.251	Calcium signaling CREB signaling Endocannabinoid neuronal synapse Glutamate receptor signaling Synaptic LTD
GRIN1	Glutamate ionotropic receptor NMDA type subunit 1	A2AI21	1.758	Calcium signaling CREB signaling Endocannabinoid neuronal synapse Glutamate receptor signaling
GRIN2B	Glutamate ionotropic receptor NMDA type subunit 2B	Q01097	1.371	Calcium signaling CREB signaling Endocannabinoid neuronal synapse Glutamate receptor signaling
GRM5	Glutamate metabotropic receptor 5	Q3UVX5	1.200	CREB signaling Endocannabinoid neuronal synapse Glutamate receptor signaling Synaptic LTD
RAP2B	RAP2B. member of RAS oncogene family	P61226	1.300	Calcium signaling CREB signaling Synaptic LTD
RYR2	Ryanodine receptor 2	E9Q401	1.251	Calcium signaling Synaptic LTD

The relative amounts (fold change, compared to WT SNs) of these proteins in Ts1Cje SNs are shown

### mGluR-LTD is enhanced in the CA1 region of Ts1Cje hippocampus

As mentioned above, the IPA analysis suggested that LTD is increased in the Ts1Cje hippocampus. It is well known that mTOR is involved in this type of plasticity [10, 12] and, remarkably, mTOR is hyperactivated in Ts1Cje hippocampus [3]. These data stimulated us to evaluate mGluR-LTD in Ts1Cje mice.

**Fig. 4:**
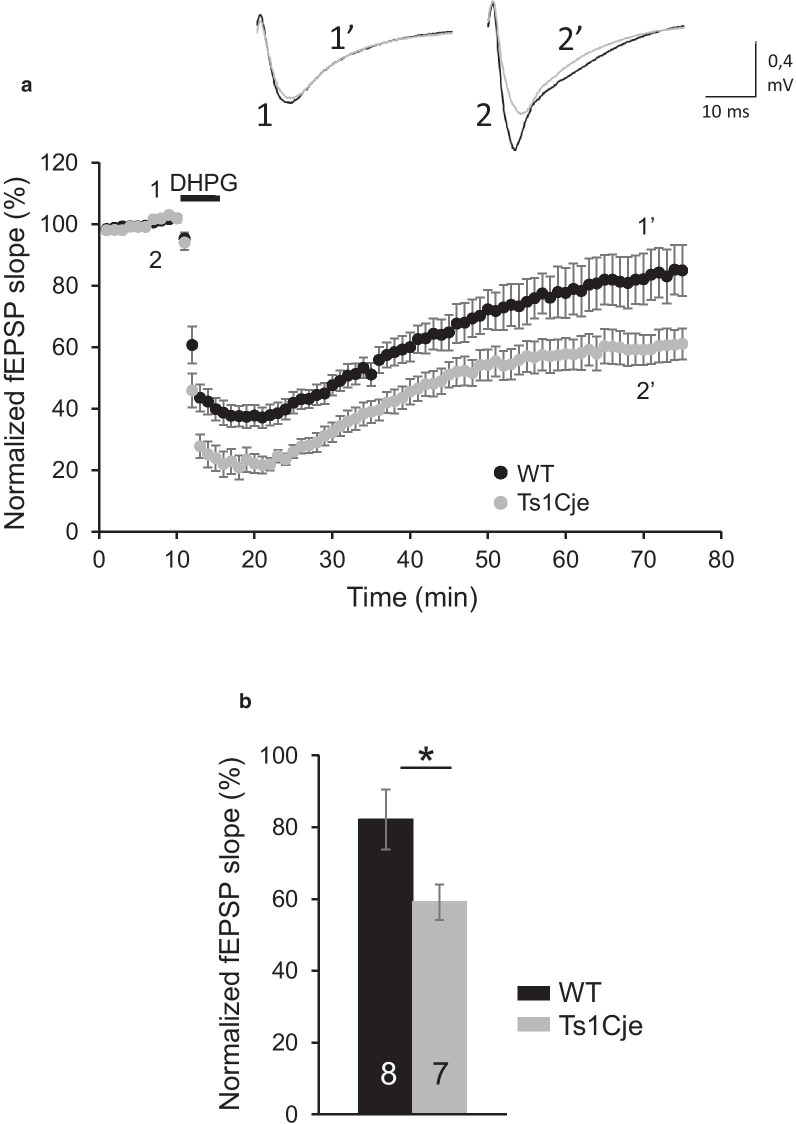
mGluR-LTD in WT and Ts1Cje hippocampal slices. **A** Time course of DHPG effects on field excitatory postsynaptic potentials (fEPSP) in WT and Ts1Cje mice. Upper insets: representative traces of a fEPSP before (1, 2) and after (1ʹ, 2ʹ) DHPG application in WT (1, 1ʹ) and Ts1Cje (2, 2ʹ) mice. The mean fEPSP slopes before DHPG perfusion between WT and Ts1Cje mice were not different. **B** Quantification of the effects depicted in **A**. The error bars represent the SEM. The number of slices for each condition is indicated in the corresponding bar (WT: 8 slices from 4 mice; Ts1Cje: 7 slices from 3 mice). p = 0.039 Student’s t-test

mGluR-LTD is experimentally induced in hippocampal slices by a brief (5 min) exposure to DHPG, a specific agonist of mGluR1/5 receptors [8]. As expected, an LTD of evoked excitatory synaptic responses was evident in WT slices after DHPG exposure (Fig. 4, 82.12 ± 8.31% of baseline, n = 8 slices from 4 mice). Remarkably, an enhanced mGluR-LTD was elicited in Ts1Cje hippocampal slices (Fig. 4, 59.12 ± 4.92% of baseline, n = 7 slices from 3 mice). In conclusion, we found that mGluR-LTD was exaggerated in the Ts1Cje CA1 hippocampal region.

**Fig. 5:**
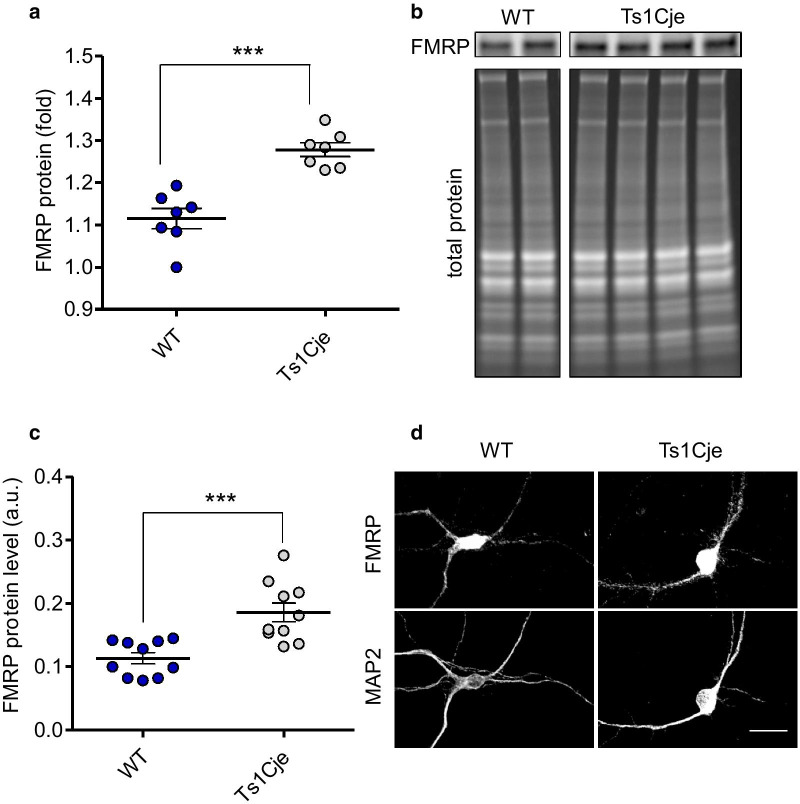
Quantification of FMRP protein in WT and Ts1Cje hippocampus. **A** Hippocampal proteins from WT and Ts1Cje mice pairs were analyzed by Western blots with an anti-FMRP antibody. The signals were normalized to the corresponding total protein loaded and the mean ± SEM values are shown (WT: 1.115 ± 0.0239; Ts1Cje: 1.278 ± 0.0162; p = 0.0006, Mann Whitney test, n = 7 animals per genotype). **B** Representative Western blot showing two WT and four Ts1Cje littermate pairs (some lanes corresponding to non-littermate animals have been removed, but both WT and Ts1Cje panels correspond to the same Western blot experiment). **C** Quantification of the relative amount of FMRP protein in dendrites of WT and Ts1Cje hippocampal neurons at DIV14. The mean pixel intensity ± SEM for FMRP immunofluorescence in dendrites is shown in arbitrary units (a.u.) (p = 0.0006, t test, n = 10 images per genotype; each image typically contained one neuron). **D** Representative gray scale confocal images from the experiment in **C**, showing FMRP and MAP2 labeling in WT and Ts1Cje neurons. Scale bar = 20 μm. Note that dendritic branching and length are reduced in Ts1Cje neurons, compared to WT controls (unpublished observations)

Enhanced mGluR-LTD is a hallmark of *FMR1* knockout mice [27]. As already mentioned, FMRP (the *FMR1* encoded protein) is a repressor of local synthesis of proteins necessary for mGluR-LTD [28–30]. Thus, we evaluated the amounts of FMRP in Ts1Cje hippocampus by Western blot and, strikingly, we found a slight, yet significant, increase of FMRP (Fig. 5). To evaluate more precisely the amount FMRP in the dendritic compartment, we performed double immunocytochemistry in primary cultures of hippocampal neurons at DIV 14 and measured the fluorescence level of FMRP labeling in MAP2-positive neurites (i.e., dendrites). As shown in Fig. 5, dendritic FMRP labeling was 1.6-fold higher in Ts1Cje neurons than in those from the WT. Hence, we must conclude that despite the higher levels of FMRP, mGluR-LTD was abnormally enhanced in Ts1Cje hippocampus.

### Prenatal treatment with rapamycin normalizes size distribution of mushroom type dendritic spines in the stratum radiatum of Ts1Cje mice

It has been proposed that LTD is a physiological correlate of spine shrinkage [13, 31, 32]. Since dendritic spine alterations have been described in DS and DS mouse models including Ts1Cje [33–35], we decided to evaluate the effect of prenatal treatment with rapamycin on dendritic spine density in postnatal Ts1Cje mice using a rapid Golgi stain method (see “Materials and methods”). We observed a reduced spine density in secondary dendrites of CA1 stratum radiatum of untreated Ts1Cje animals, compared to WT controls (WT: 0.640 ± 0.031 spines/μm, n = 50; Ts1Cje: 0.523 ± 0.035 spines/μm, n = 26; t-Student test p-value = 0.015). However, prenatal treatment with rapamycin produced no significant effect on spine density in either group (WT RAPA: 0.666 ± 0.029 spines/μm, n = 27; Ts1Cje RAPA: 0.572 ± 0.021 spines/μm, n = 31).

**Fig. 6:**
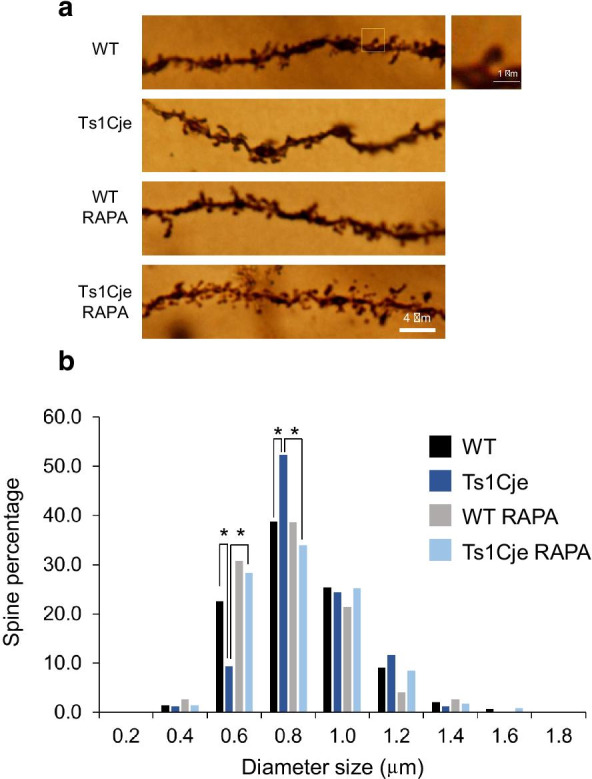
Frequency distribution of mushroom spines clustered by diameter size in CA1 stratum radiatum of untreated animals (WT and Ts1Cje) or mice treated with rapamycin prenatally (WT RAPA and Ts1Cje RAPA). **A** Representative images of Golgi staining for WT, Ts1Cje, WT RAPA and Ts1Cje RAPA mice; a typical mushroom spine is also shown. **B** Distribution of mushroom spines clustered by diameter size. Frequencies are shown as percentages. Center of the first and last bins in the histogram were automatically fixed (using GraphPad Prism software), and bin wide was set to 0.2 μm. The Z-score for two population proportions was calculated between WT vs. Ts1Cje, WT vs. WT RAPA and Ts1Cje vs. Ts1Cje RAPA for each histogram interval. Statistically significant p-values were obtained when comparing WT vs. Ts1Cje, and Ts1Cje vs. Ts1Cje RAPA, (as indicated with asterisks) in the following cases: 0.5 to 0.7 μm interval (bin center 0.6 μm): WT vs. Ts1Cje p-value = 0.011, Ts1Cje vs. Ts1Cje RAPA p-value < 0.001; 0.7 to 0.9 μm interval (bin center 0.8 μm): WT vs. Ts1Cje p-value = 0.045, Ts1Cje vs. Ts1Cje RAPA p-value = 0.002

It has been shown that the CA1 spines susceptible to undergo mGluR-LTD are large-head mushroom spines that contain endoplasmic reticulum (ER) and often harbor a spine apparatus, whereas spines without ER are refractory to this plasticity [36]. Thus, we decided to assess the proportion of filopodia, stubby and mushroom shape spines but we found no significant differences in the proportion of the different morphology categories among the experimental groups (Table 3). Howewer, we found that the proportion of 0.5–0.7 μm mushroom spines was significantly reduced in Ts1Cje animals, compared to WT, and recovered in Ts1Cje RAPA mice, whereas the percentage of 0.7–0.9 μm mushroom spines was significantly increased in Ts1Cje mice and restored in Ts1Cje RAPA animals (Fig. 6 and Additional file 3: Table S8). Thus, prenatal treatment with rapamycin normalizes the size distribution of mushroom type dendritic spines in Ts1Cje stratum radiatum neurons.

**Table 3:** Dendritic spine morphology comparison

Spine morphology	WT (n = 248)	Ts1Cje (n = 151)	WT RAPA (n = 143)	Ts1Cje RAPA (n = 635)
Filopodium	14.9	13.2	22.4	19.7
Stubby	27.8	29.8	25.2	26.5
Mushroom	57.3	57.0	52.4	53.9

The percentages of filopodia, stubby and mushroom spines present in secondary dendrites of apical stratum radiatum CA1 neurons in WT, Ts1Cje, and prenatally rapamycin-treated WT and Ts1Cje mice are indicated. Number of mice used per condition was 2, except for WT RAPA (a single animal). Total number of spines (n) is also indicated for each experimental group

### Prenatal treatment with rapamycin reduces mGluR-LTD in Ts1Cje hippocampus

**Fig. 7:**
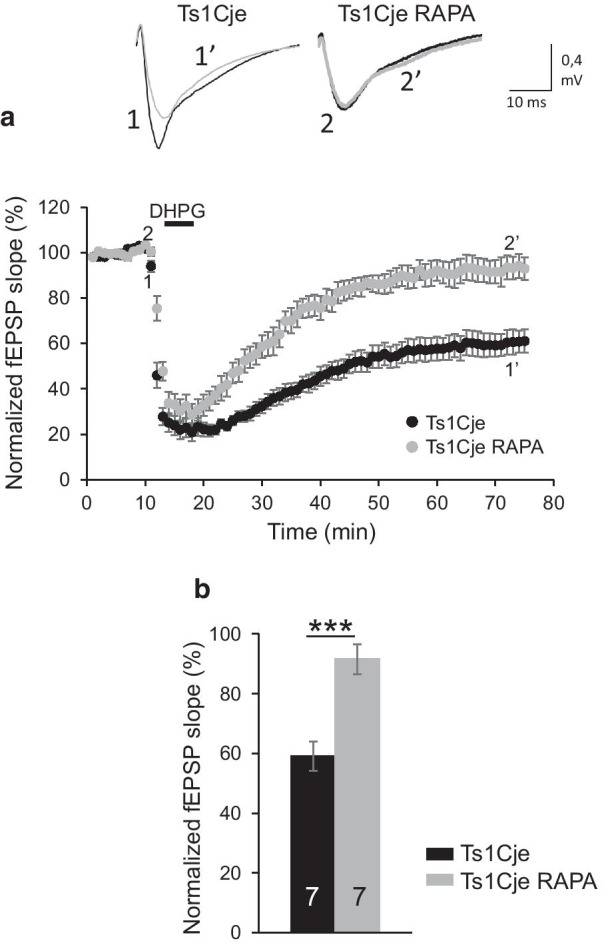
mGluR-LTD in hippocampal slices of Ts1Cje mice treated with rapamycin prenatally. **A** Time course of DHPG effects on field excitatory postsynaptic potentials (fEPSP) in Ts1Cje mice treated with rapamycin prenatally (Ts1Cje RAPA) and control Ts1Cje mice (same data as in Fig. 4). Upper insets: representative traces of a fEPSP before (1, 2) and after (1ʹ, 2ʹ) DHPG application in Ts1Cje (1, 1ʹ; same data as in Fig. 4) and Ts1Cje RAPA (2, 2ʹ) mice. The mean fEPSP slopes before DHPG perfusion between Ts1Cje and Ts1Cje RAPA mice were not different. **B** Quantification of the effects depicted in **A**. The error bars represent the SEM. The number of slices for each condition is indicated in the corresponding bar (Ts1Cje: 7 slices from 3 mice; Ts1Cje RAPA: 7 slices from 4 mice). p < 0.001 Student’s t-test

In order to determine if the normalization of mushroom spine size distribution observed in Ts1Cje treated prenatally with rapamycin (Fig. 6) correlated with an effect on mGluR-LTD, we evaluated this plasticity in hippocampal slices of postnatal Ts1Cje mice treated prenatally with rapamycin (see “Materials and methods”). Remarkably, as shown in Fig. 7, mGluR-LTD was reduced in Ts1Cje RAPA mice (91.55 ± 5.23% of baseline, n = 7 slices from 4 mice) compared to Ts1Cje, and showed values similar to those of WT.

## Discussion

We have previously shown that the mTOR pathway is hyperactivated in Ts1Cje hippocampus [3]. Afterwards, other groups reported mTOR hyperactivation in postmortem DS brain [4, 5]. We have also found that rapamycin restored both BDNF-LTP and the persistence of LTM in the Barnes maze [7].

We have here presented the characterization of the Ts1Cje hippocampal synaptic proteome, and we have identified several affected pathways that could account for plasticity and/or memory deficits of Ts1Cje mice. Two main functions were predicted to be altered in Ts1Cje hippocampus: mitochondrial function and synaptic plasticity (including LTD).

Mitochondrial dysfunction and increased oxidative stress have been previously found in the Ts1Cje brain [37]. In fact, altered mitochondrial function has long been associated with DS [23–25]. It has been recently found damaged mitochondria linked to increased oxidative stress, reduced mitophagy and reduced autophagy, together with mTOR hyperactivation in fibroblasts from DS patients [26]. The role of mTORC1 as regulator of both general autophagy and mitophagy induction after oxidative phosphorylation uncoupling is well established [38]. Accordingly, pharmacological inhibition of mTOR using AZD8055, which inhibits both mTORC1 and mTORC2 [39], restored autophagy and mitophagy in DS fibroblasts [26]. As mentioned before, we previously demonstrated hyperactivation of mTOR in the Ts1Cje hippocampus [3]. Accordingly, we have here observed that autophagy is reduced, which could be related to the mitochondrial dysfunction that we detected in the proteomic analysis of Ts1Cje SNs, and to the increased levels of the mitochondrial mass marker TOM20 we measured in Ts1Cje adult hippocampus (Fig. 3).

Regarding synaptic plasticity, we found that mGluR-LTD is enhanced in Ts1Cje hippocampus. Many forms of synaptic plasticity have been characterized in the Ts1Cje and other DS mouse models [7, 35, 40]. mGluR-LTD has been previously studied in the Ts65Dn model but, in contrast to our results, a normal hippocampal mGluR-LTD, compared to WT, was found [41]. Nevertheless, there are important differences between the Ts65Dn and Ts1Cje models that could explain this apparent contradiction. Thus, additional DS non-related trisomic genes exist in Ts65Dn [42]. The fact that 6–8 month-old mice were used in the Scott-McKean and Costa study while we used P21–30 mice could also explain the different results since the mechanisms of mGluR-LTD seem to be developmentally regulated [43].

mGluR-LTD is triggered by activation of group I mGluR (i.e., mGluR1/5) and relies on protein translation [8]. Two main signaling cascades that regulate protein synthesis are engaged following mGluR1/5 stimulation: mTOR and ERK. Both pathways can stimulate cap-dependent translation at the initiation level. The relative contribution of these cascades to the protein synthesis necessary for mGluR-LTD is nevertheless unclear (for a review see [16]). Interestingly, it has been recently shown that mTORC2, but not mTORC1, is required for mGluR-LTD [12]. mTORC2 regulates actin cytoskeleton and, in fact, actin polymerization–depolymerization inhibition abolishes mGluR-LTD [13]. In any case, it seems clear that in wild-type conditions, rapid synthesis and degradation of FMRP is necessary for mGluR-LTD [17], which leads to transient local translation of key proteins involved in AMPAR internalization (i.e. GluA1 endocytosis), such as Arc/Arg3.1 [28, 30, 44]. Thus, after mGluR1/5 stimulation, FMRP is rapidly (< 1 min) dephosphorylated by protein phosphatase 2A, and then (1–5 min) re-phosphorylated by p70S6K [45], a kinase that belongs to the mTOR pathway. Dephosphorylated FMRP is associated with polyribosomes, and phosphorylated FMRP with stalled (non-translating) ribosomes [46]. Thus, the short period after mGluR1/5 in which FMPR is dephosphorylated permits the translation of the FMRP-regulated proteins involved in mGluR-LTD [45].

Remarkably, and similarly to our results in Ts1Cje mice, it is well established that mGluR-LTD is exaggerated in FMRP knockout (KO) mice [27], a model for Fragile X. However, Fragile X is due to loss of expression of FMRP and, in contrast, we found higher levels of FMPR in the Ts1Cje hippocampus, strongly suggesting a different cause for the enhanced hippocampal mGluR-LTD in trisomic mice. From the list of proteomic changes involved in LTD identified in Ts1Cje SNs, the increased level of mGluR5 (indicated as GMR5 in Table 2) is of particular interest, since it is the main receptor that mediates mGluR-LTD. Thus, the 1.2-fold increase of mGluR5 in Ts1Cje SNs we detected could collaborate to the exaggerated mGluR-LTD we observed. On the other hand, it has been shown that the spines susceptible to undergo mGluR-LTD in the CA1 constitute a particular subpopulation of large-head mushroom spines with ER and spine apparatus, whereas spines without ER are refractory to mGluR-LTD [36]. We have here shown that the percentage of mushroom spines with a 0.7–0.9 μm size was notably increased in Ts1Cje CA1 dendrites, compared to WT (Fig. 5). Although we are not sure if this subpopulation corresponds to that described in ref. [36], it is tempting to speculate that exaggerated mGluR-LTD in Ts1Cje hippocampus could be due to a higher proportion of ER-containing spines, susceptible to undergo mGluR-LTD. Excitingly, rapamycin treatment of pregnant dams normalized the referred mushroom spine phenotype and reduced the mGluR-LTD in the Ts1Cje offspring.

mGluR stimulation leads to local inositol trisphosphate (IP3) receptor activation and calcium release from the ER [36]. Moreover, ryanodine receptors (RyRs) are particularly abundant in the spine apparatus [47], an ER membrane specialization of stacked discs, which plays roles in local translation and calcium signaling [48]. mGluR-LTD induces trafficking from the ER to the synapse of GluA2, an AMPAR subunit that renders the receptor impermeable to calcium. This trafficking depends on IP3 and RyR-mediated calcium release, and translation [49, 50]. Interestingly, IPA of the Ts1Cje hippocampal SNs proteomic data predicted increased calcium signaling due to higher levels of RyR2, glutamate receptor subunits (including GluA2, GluA4, GluN1, GluN2B), plasma membrane calcium ATPases, and calcium voltage-gated channel subunits (Table 2). We think that the increased levels of these proteins could be a consequence of the higher percentage of the 0.7–0.9 μm mushroom spines in Ts1Cje hippocampus. Interestingly, the morphology of dendritic spines appeared among the functions that IPA predicted to be affected in Ts1Cje (not shown), based on the altered expression of several proteins, including catenin alpha 2 (CTNNA2). Remarkably, it has been shown that the mutation of CTNNA2 induces spine elongation in hippocampal neurons [51]. Conversely, we hypothesize that the higher level of CTNNA2 we found in Ts1Cje SNs (Additional File 2: Table S5) could provoke spine enlargement. Nevertheless, it should be noted that adult animals were used for proteomics while juvenile mice were used for electrophysiology experiments and spine morphology assessment. Thus, these correlations should be taken with precaution.

In summary, the molecular mechanism behind the mushroom spine morphologic phenotype in Ts1Cje hippocampal dendrites is unknown. Nevertheless, since it is recovered by rapamycin, mTOR signaling should be involved. We have previously found increased levels of both phospho-S6 (Ser235/236), a redout of mTORC1, and phospho-Akt (Ser473), a redout of mTORC2, in dendrites of Ts1Cje hippocampal neurons [3]. Accordingly, either mTORC1 or mTORC2 (or both) could be involved. Interestingly, RICTOR, a canonical component of mTORC2, interacts with Tiam1, a Rac-1 specific guanine nucleotide exchange factor, to regulate actin polymerization [52]. Tiam1 is encoded in the human chromosome 21 and it is in trisomy in Ts1Cje mice. Thus, Tiam1 overexpression in addition to mTORC2 hyperactivation could be relevant for the spine phenotype.

In conclusion, we have here shown abnormal, exaggerated hippocampal mGluR-LTD in Ts1Cje mice, which correlates with an increased proportion of 0.7–0.9 μm mushroom spines in CA1 dendrites. Although the precise molecular/cellular mechanisms for these phenotypes remain to be elucidated, prenatal treatment with rapamycin restored both morphologic and functional phenotypes, highlighting the therapeutic potential of rapamycin/rapalogs for correcting synaptic defaults in DS.

## Supplementary Information

**Additional file 1: Table S1**. List of proteins detected by tandem mass spectrometry iTRAQ in technical duplicates of WT and Ts1Cje synaptoneurosomes (SNs). Quantification is referred to one of the WT samples. **Table S2.** Same data as in Table S1 after elimination of proteins identified by only one unique peptide. **Table S3.** Geometric mean of the relative protein amount in Ts1Cje SNs, after elimination from Table S2 of proteins not detected in all the samples, as well as technical replicates with CV% > 30%. **Table S4.** Complete hierarchy of PANTHER Go-Slim Cellular Component proteins overrepresented and underrepresented in WT and Ts1Cje SNs.

**Additional file 2: Table S5.** List of proteins affected in Ts1Cje SNs. **Table S6.** List of canonical pathways affected in Ts1Cje SNs. **Table S7.** Z-score analysis of canonical pathways in Ts1Cje SNs.

**Additional file 3: Table S8.** Number of spines belonging to each category (size) included in Fig. 6.

## Data Availability

All data generated or analysed during this study are included in this published article and its additional information files. A preprint of this manuscript, including datasets, is available at: https://doi.org/10.1101/2020.04.08.032029.

## Abbreviations

ACSFs: Artificial cerebrospinal fluid with partial substitution of Na with sucrose
AMPAR: AMPA receptor
Arc/Arg3.1: Activity Regulated Cytoskeleton-associated protein
a.u.: Arbitrary units
BDNF: Brain Derived Neurotrophic Factor
CTNNA2: Catenin alpha 2
DHPG: (S)-3,5-Dihydroxyphenylglycine
DIV: Days in vitro
DS: Down syndrome
ER: Endoplasmic reticulum
ERK: Extracellular signal-regulated kinase
FDR: False Discovery Rate
fEPSP: Field excitatory postsynaptic potential
FMRP: Fragile X mental retardation protein
IP: Inositol trisphosphate
IPA: Ingenuity Pathway Analysis
iTRAQ: Isobaric tag for relative and absolute quantitation
KO: Knockout
LC3B-II: Microtubule-associated protein 1A/1B-light chain 3
LTD: Long term depression
LTM: Long-term memory
LTP: Long-Term Potentiation
MAP1B: Microtubule Associated Protein 1B
mGluR-LTD: Metabotropic Glutamate Receptor-Long Term Depression
MMU: Murine chromosome
mTOR: Mammalian target of rapamycin
N/A: Not applicable
NEO: Neomycine
OPHN1: Oligophrenin-1
P: Postnatal day
PANTHER: Protein Analysis Through Evolutionary Relationships
PBS: Phosphate-buffered saline
PFA: Paraformaldehyde
PVDF: Polyvinylidene difluoride
RAPTOR: Regulatory Associated Protein of mTOR
RICTOR: Rapamycin-Insensitive Companion of TOR
RRID: Research Resource Identifier
RT: Room temperature
RyRs: Ryanodine receptors
SEM: Standard error of the mean
SNs: Synaptoneurosomes
STEP: Striatal-Enriched Protein Phosphatase
TOM20: Translocase of Outer Mitochondrial membrane 20
WT: Wild-type
